# Isolation and identification of new bacterial stains producing equol from *Pueraria lobata* extract fermentation

**DOI:** 10.1371/journal.pone.0192490

**Published:** 2018-02-15

**Authors:** Jeong Eun Kwon, Jaewon Lim, Inhye Kim, Donghyuk Kim, Se Chan Kang

**Affiliations:** College of Life Science, Kyung Hee University, Yongin, Republic of Korea; Babasaheb Bhimrao Ambedkar University, INDIA

## Abstract

Equol is a nonsteroidal estrogen that is produced by intestinal bacterial metabolism. Equol and equol-producing bacteria have been extensively investigated with soybean-based materials under anaerobic condition. In this study, an under-appreciated plant material, *Pueraria lobata*, was used to find new bacterial strains that produce equol under aerobic conditions. Three new intestinal bacteria, CS1, CS2, and CS3, were isolated, and internal transcribed spacer analysis revealed that belonging to genus *Pediococcus* and *Lactobacillus*. HPLC analysis showed that these strains produced equol or its related intermediates when fermenting *P*. *lobata* extract. In comparison to fermentation of *P*. *lobata* extract, soybean germ extract was also fermented. While the isolated strains did not produce equol in this extract, they produced other equol-related precursors. To test the modularity effect of these fermentation mixtures with the newly isolated bacteria, MCF-7 cell proliferation assay was performed, which showed that all extracts fermented with those strains has a modularity effect. Fermenting *P*. *lobata* extract with strain CS1 demonstrated the best modularity effect.

## Introduction

Equol (4’,7-isoflavandiol) is an isoflavandiol commonly metabolized from daidzein and is a nonsteroidal estrogen of the isoflavone class [1]. It has been well established that equol is exclusively produced by intestinal bacterial metabolism, and its clinical importance for estrogenic activity and affinity for estrogen receptors have been studied [2]. Equol can be metabolized from biotransformation of the phytoestrogens, daidzein and genistein, which are isoflavones. These isoflavones are particularly prevalent in soybean foods [3], thus bioconversion of precursor isoflavones to equol or related intermediates by bacterial strains has been investigated in the context of soybean products. Such intestinal bacterial strains have been successfully isolated from human and animal feces [4–6] and a number of bacterial strains have been isolated and further characterized [7]. Fermentation to produce equol or related intermediates from soybean-based materials has been generally performed under anaerobic conditions, similar to the gastrointestinal environment where these bacterial strains dwell and produce equol.

*Pueraria lobata*, which has been more commonly called arrowroot or kudzu, is a perennial legume that is widely distributed in East Asia and tropical and subtropical countries [8]. Traditionally, it has been used medicinally in Korea, Japan, and China, and the roots have been shown to suppress alcohol intake, to reduce blood pressure, and to prevent osteoporosis [9]. *In vitro* culture assay revealed that certain *P*. *lobata* plant parts contain a significant amount of daidzein glycosides, such as puerarin and daidzin. The concentrations were higher in the root callus than in the callus from the leaves and stem segments [9]. Thus, fermenting *P*. *lobata* root extract could be reasonably expected to produce equol or its related intermediates.

In this study an under-appreciated plant material, *P*. *lobata* root extract, was used to identify new bacterial strains that can produce equol from isoflavones. In addition, aerobic culture has benefits over anaerobic culture in terms of cost and resource use. Thus, aerobic culture was used to identify new equol-producing bacterial strains.

## Materials and methods

### *P*. *lobata* and Soybean germ extract preparation

*Pueraria lobata* (Willd.) Ohwi roots were provided by Professor Tae-Ho Park from the greenhouse at Daegu University (Daegu, South Korea) and soybean germ were purchased from Dr Chung’s Food Co., Ltd. (Seoul, Korea).

Since the total amount of daidzein glycosides, such as puerarin and daidzin, is highest in the *P*. *lobata* root callus [9], root segments were used in the extract for equol bioconversion. Using the root extract of *P*. *lobata* for the bacterial cultures necessitated lyophilization of the extract contents. *Pueraria lobata* (Willd.) Ohwi roots were chopped into small pieces and extracted three times in 20% ethanol for 24 hr each at room temperature. Various ethanol concentrations were tested, and 20% gave the best yield. The extract was subsequently filtered to remove any particulates and was concentrated under vacuum at 50°C. Then, the concentrated crude extract was lyophilized to obtain a powder and stored at -20°C for further experiments (20.5% yield).

One kilogram of soybean was mixed with 10 L of 30% ethanol and then were extracted at 80 °C for 3 hr. The mixture was subsequently filtered to remove any particulate materials and vacuum concentrated at 50 °C. The concentrated crude extract was lyophilized to obtain a powder that was stored at -70 °C for further experiments.

### Bacterial culture and media preparation

Fecal suspensions were plated onto several different media plates: Gifu Anaerobic Medium (GAM), Trypticase Soy Agar (TSA), Eosin Methylene Blue (EMA), Reinforced Clostridial Media (RCM), Brain heart infusion (BHI), and de Man, Rogosa and Sharpe (MRS). To isolate equol-producing strains under aerobic conditions, colonies from the plates were further cultured in liquid GAM medium (Becton, Dickinson and Company, New Jersey, USA) containing 2% *P*. *lobata* extract (w/v) or 2% soybean germ extract (w/v) at 28°C or 37°C for 72 hr with agitation. GAM medium was chosen because the candidate strains grew fastest in that medium.

We did not obtain ethics approval for the study, because ethics approval was not necessary. Particularly in the clinical diagnosis, the human-derived substances refer to blood, organ tissue, urine, etc. Feces used in this study were not stored as human-derived materials nor used in the clinical diagnosis, rather just used to isolate microorganisms. Thus, IRB was not required.

### Thin layer chromatography for equol detection

TLC experiments to screen for isoflavones were performed with the following method from a previous publication [10] with slight modifications. In brief, bacterial culture supernatant was transferred to RP-18 silica gel TLC plates (20 × 20 cm, Merck, 60 F, 254 nm, Darmstadt, Germany) with a solvent system of acetonitrile: water: acetic acid (60:40:1). Isoflavones on TLC were visualized with a UV transilluminator (321 nm) by spraying with anisaldehyde reagent. Pure equol (Cayman Chemical, Michigan, USA) was used as a positive control in the TLC experiments.

### High-performance liquid chromatography (HPLC) analysis

The HPLC system was a DGU-20A3R HPLC apparatus (Shimadzu, Japan) with a photodiode array detector (SPD-M20A, Shimadzu, Japan). A Skypack C18 column (SK chemical, Seoul, Korea, 4.6 × 250 mm, 5 μm) was used to analyze all samples. The mobile phases were water: acetic acid (100:1, solvent system A) and water: acetonitrile: acetic acid (50:50:1, solvent system B) in gradient mode.

### Strain identification by sequencing the ribosome 16S internal transcribed spacer (ITS) regions

Bacterial strains CS1, CS2, and CS3, were cultured at 28°C in an aerobic incubator for 16 hr and were collected by centrifugation at 10,000 × g for 3 min. Genomic DNA was extracted with a genomic DNA prep kit (Solgent, Daejeon, Korea) by following the manufacturer’s protocol. The 16S rRNA gene was amplified by polymerase chain reaction (PCR) with primers described previously [11]. The PCR mixture (20 *μ*L) contained 2× Taq PCR premix (Qiagen), 10 pM each primer (S2 Table), double distilled water, and 1 *μ*L of genomic DNA. The PCR conditions were as follows: 94°C for 2 min; 30 cycles of 94°C for 10 s, 45°C for 20 s, and 72°C for 60 s; and 72°C for 5 min. The PCR product was mixed with 6× gel loading dye and subjected to electrophoresis on a 0.7% agarose gel to confirm the PCR product. The amplified product was excised from the gel and purified with a QIAquick Gel Extraction Kit (Qiagen).

### Phylogenetic tree analysis

A phylogenetic tree was constructed by using internal transcribed spacer (ITS) sequences with the ETE toolkit [12]. Of 29 published equol-producing strains [7], only one, *Eggerthella* sp. YY7918, has a complete genome sequence [13]. In addition, 14 bacterial species that the other equol-producing strains belong to and that have genome sequences at NCBI were used to prepare ITS sequences between the 16S and 23S rRNA genes. Each number is expressed as a percentage of 100 replications. The scale bar represents genetic distance (the number of mutations/evolutionary events between species since their divergence).

### E-screen assay

To evaluate estrogenic-like effects, a sensitive E-screen assay by cell proliferation was performed with human ER-positive breast adenocarcinoma cells (MCF-7). This assay measures the proliferation of MCF-7 cells as an indirect proxy of the estrogenicity of compounds. The technique was adopted from the previous study [14]. In brief, trypsinized MCF-7 cells were seeded in 24-well plates at an initial concentration of 20,000 cells per well in 10% FBS in RPMI. To permit their adhesion, cells were incubated for 24 h (37 °C, 5% CO_2_), and were washed with phosphate-buffered saline (PBS). The Serum Replacement 2 (0.5×) supplemented phenol red-free RPMI was substituted for the seeding medium. The synthetic estrogen or the fermentation mixture were added to the experimental medium at concentrations from 0.1 nM to 1 μM. After 144 hr, the medium was removed from wells to stop the assay. Cells were fixed and were stained with sulforhodamine-B (SRB). Bound dye was solubilized with 10 mM Tris base (pH 10.5) in a shaker. At last, aliquots were read in a Biotek EL800 Multiscan apparatus (Winoosky, USA) at 510 nm. The measured estrogenic activity was shown as mean ± standard error of mean of the proliferative effect (PE).

### Quantitative reverse transcription polymerase chain reaction

Human breast ER+ cancer, MCF-7 Cells were treated with samples for 48 hours. Total RNA from cells was extracted with TRIzol reagent (Invitrogen) following the manufacturer’s instruction, and complementary DNA was synthesized using Moloney murine leukemia virus reverse transcriptase with random primers. The expression levels of genes were determined by quantitative PCR. Complementary DNA was generated with PrimeScriptTM 1st strand cDNA Synthesis Kit (Takara) according to the manufacturer’s protocol. qRT-PCR was performed using SYBR Premix Ex Taq^™^ (TaKaRa).

## Results

### Isolation of human intestinal bacteria capable of producing equol

To isolate possible bacterial strains that might be able to convert daidzein glycosides to equol, 14 healthy volunteers (6 females and 8 males, 25–41 years old) consumed 100 mL of *P*. *lobata* extract (1:10 extract in water) for 14 days. Fresh fecal specimens were suspended in 1 ml sterile phosphate buffer saline (PBS) solution. Then 0.1 ml of fecal suspension was plated onto various media, such as GAM, TSA, EMA, RCM, BHI, and MRS. A number of colonies were selected from the plates and were further cultured under aerobic conditions in liquid GAM medium containing 2% (w/v) *P*. *lobata* extract at 28°C or 37°C. GAM medium was used because the isolated strains grew fastest in GAM medium under aerobic conditions. After a 24-hour incubation, the presence of equol in samples was assessed by thin layer chromatography (TLC) experiments.

TLC was used to screen isoflavones by following a previously published method [10] with a slight modification. Of the approximately 200 colonies from plates of various media, 3 had a TLC band indicating the presence of equol molecules in the culture. These three bacterial strains were used for further analyses and are referenced as CS1, CS2, and CS3.

### Characterization of production capability of equol and related intermediates

**Fig 1:**
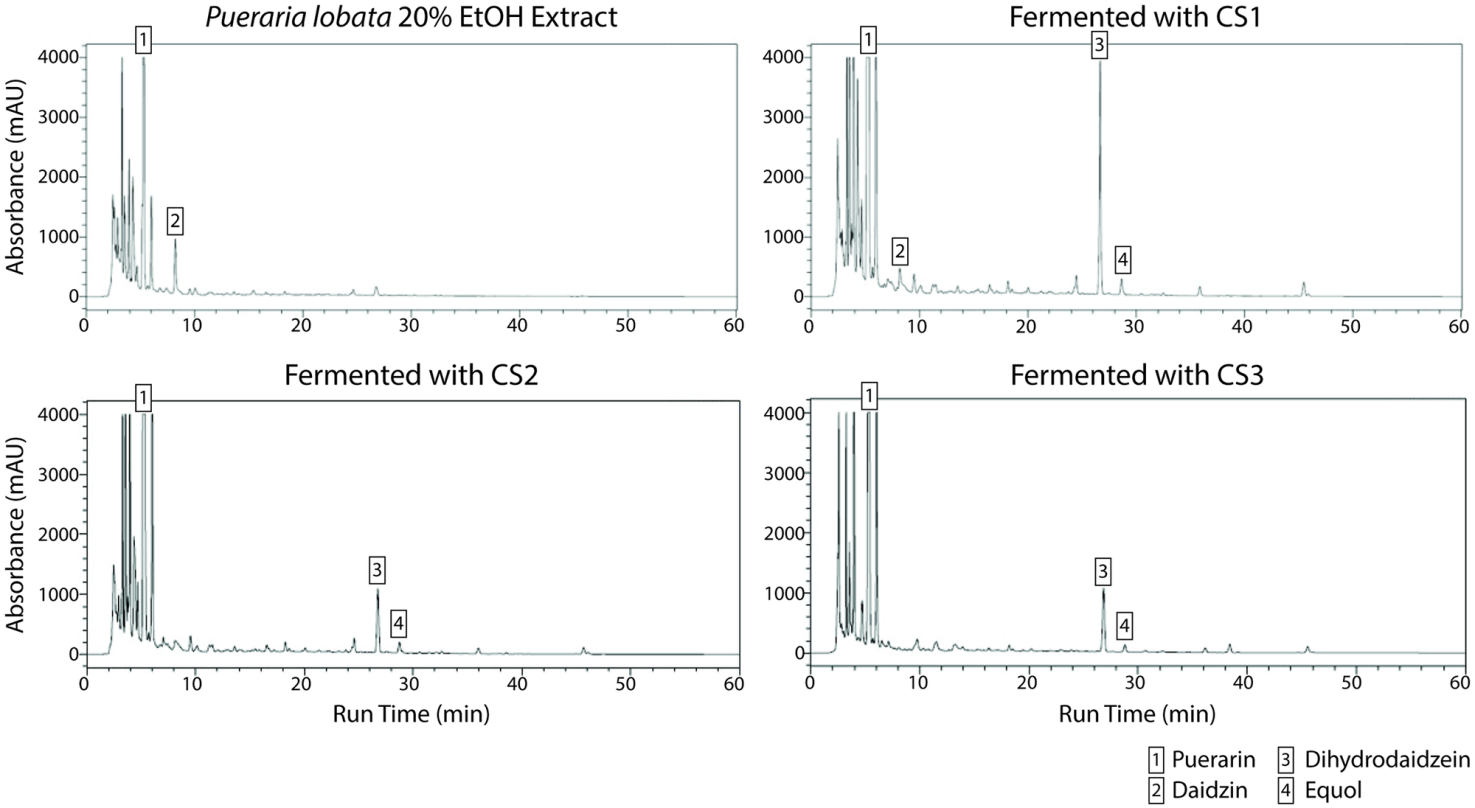
Analysis of equol-related components in *P*. *lobata* extract medium before and after fermentation. HPLC analysis of puerarin, dihydrodiadzein, daidzin, and equol in *P*. *lobata* extract medium before and after fermentation.

To measure the consumption of precursor molecules and the production of equol or related products, the three newly isolated bacterial strains, CS1, CS2, and CS3 in Fig 1, and Table 1, were further examined by HPLC. The HPLC experiments showed that a 20% ethanol extract of *P*. *lobata* had a high amount of puerarin and a decent amount of daidzin (Fig 1). After fermentation with the three bacterial strains, the concentrations of puerarin and daidzin, which are equol precursors, decreased, while the dihydrodiadzein (DHD, an equol precursor) and equol concentrations increased (Fig 1). This result indicates that all three bacterial strains contributed in bioconversion of the precursor molecules into DHD or equol.

**Table 1:** Concentration of puerarin, daidzin, DHD, and equol in *P*. *lobata* extract medium before and after fermentation. (ND: not detected).

	Extract (mg/g)	CS1 (mg/g)	CS2 (mg/g)	CS3 (mg/g)
Puerarin	195	194	192	201
Daidzin	78.1	10.3	N/D	N/D
Dihydrodaidzein	N/D	196	82	84
Equol	N/D	12.3	7.4	7.9

The DHD and equol concentrations were measured by lyophilizing culture samples before and after fermentation with the newly isolated bacterial strains (Table 1). Strain CS1 produced the most abundant amount of equol and its precursor, DHD. The other two strains, CS2 and CS3, also produced 60.2% and 64.2% as much equol as CS1 and 41.8% and 42.9% as much DHD as CS1.

In summary, all of the three strains proved the ability to consume daidzein glycosides and to produce equol and its precursor, DHD. However, strain CS1 was the most efficient strain in converting daidzein glycosides to equol and DHD.

### Identification of equol-producing strains

Isolation of the equol-producing bacterial strains entailed further identification of those strains. Bacterial strains can be identified by biochemical, morphological, cultural, molecular, and physiological properties. Sequence-based analysis of 16S rRNA, particularly the internal transcribed spacer (ITS), has been widely used to identify various organisms including bacteria [15], eukaryotic microorganisms [16], fungi [17], plants [18], and animals [19]. Thus, the ribosomal ITS regions from the three bacterial strains were amplified by PCR and were sequenced (S1 Table).

ITS sequences were submitted for BLAST analysis to identify bacterial strains (Table 2). Strain CS1 had an identical ITS sequence to *Pediococcus pentosaceus*, CS2 had an identical ITS sequence to *Lactobacillus casei* or *Lactobacillus paracasei*, and CS3 had an identical ITS sequence to *Lactobacillus sakei* or *Lactobacillus graminis*. Those three strains belong to a genus of gram-positive lactic acid bacteria in the Lactobacillaceae family. Multiple intestinal bacteria in a wide range of families have been identified to biotransform isoflavones to equol or related intermediates [7], and a few of these strains belong to genus *Lactobacillus* [20, 21].

**Table 2:** Identification of equol-producing strains by ITS sequence analysis.

Strain	Species	Query Coverage	Sequence Identity
CS1	*Pediococcus pentosaceus*	100%	99%
CS2	*Lactobacillus casei*	100%	99%
	*Lactobacillus paracasei*	100%	99%
CS3	*Lactobacillus sakei*	100%	99%
	*Lactobacillus graminis*	100%	99%

**Fig 2:**
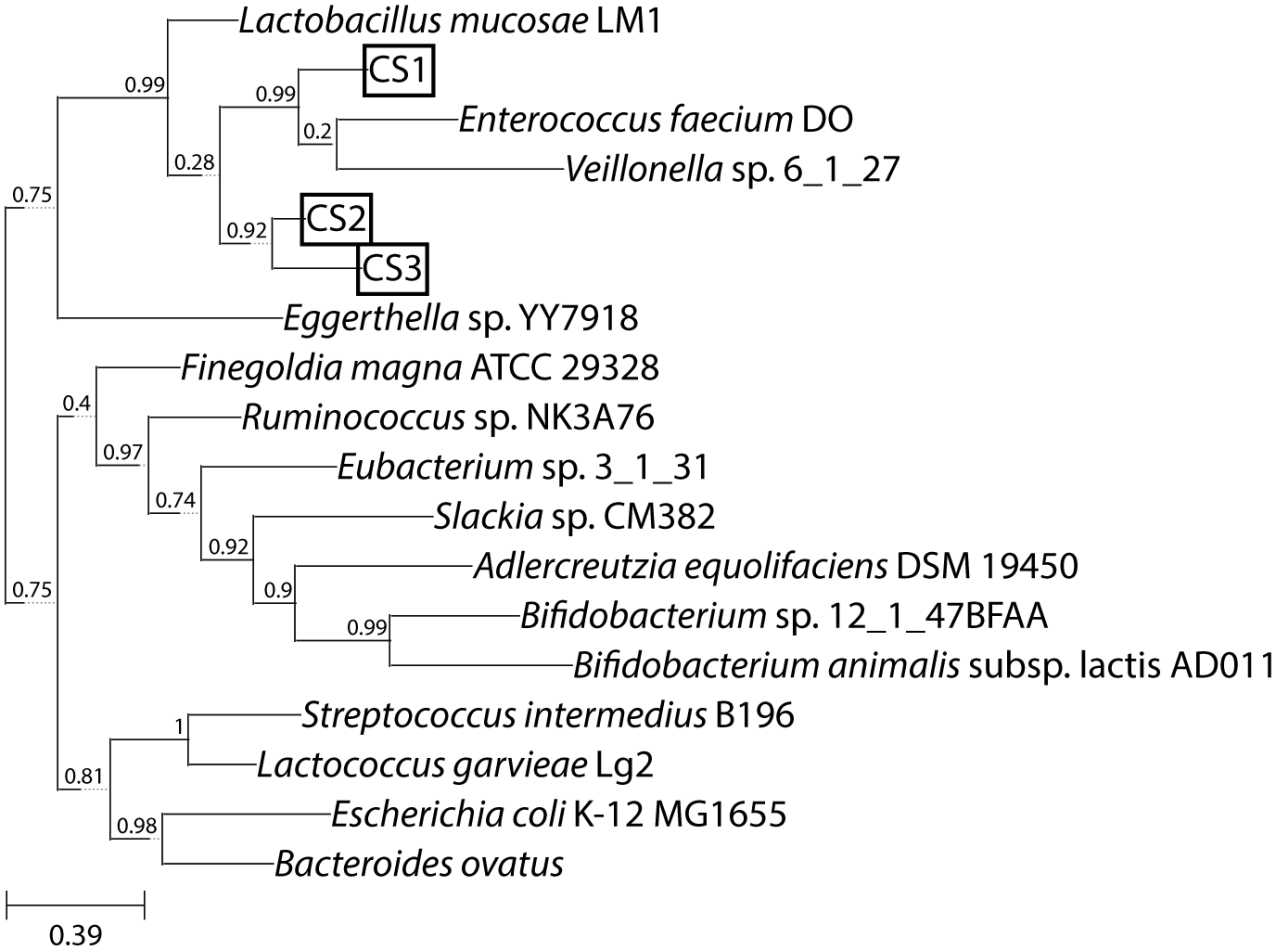
Phylogenetic tree of the newly isolated strains and other bacterial strains to which the previously reported equol-producing strains belong.

The bacterial strain, *Eggerthella* sp. YY7918, has a proven ability to produce equol [22] and has a complete genome sequence [13]. Thus, the ITS sequence was prepared from the genome sequence of *Eggerthella* sp. YY7918. Among 29 equol-producing strains [7], 14 bacterial strains belong to bacterial species or genus that have the published genome sequences at NCBI, thus ITS sequences were prepared from those genome sequences as well. Phylogenetic tree analysis showed that strains CS2 and CS3 are quite close to each other, and strain CS1 is also close to the other two strains. All three strains are relatively close to *Lactobacillus mucosae* and *Eggerthella* sp. YY7918. (Fig 2)

Summarily the ITS analysis showed that the strain CS1 belongs to *Pediococcus pentosaceus*, and the strains CS2 and CS3 belong to *Lactobacillus*. These species were known to be closely related to other published equol-producing bacteria.

### Fermentation of soybean germ extract

Equol production has been most extensively investigated in the context of soy-based diets, and the precursor molecules to equol and DHD, daidzein glycosides have been considered to be soy isoflavones [7]. Thus, it became of interests to see if the newly isolated bacteria, which could produce equol and related intermediates from *P*. *lobata* root extract, can also produce equol from soy-based extract.

**Fig 3:**
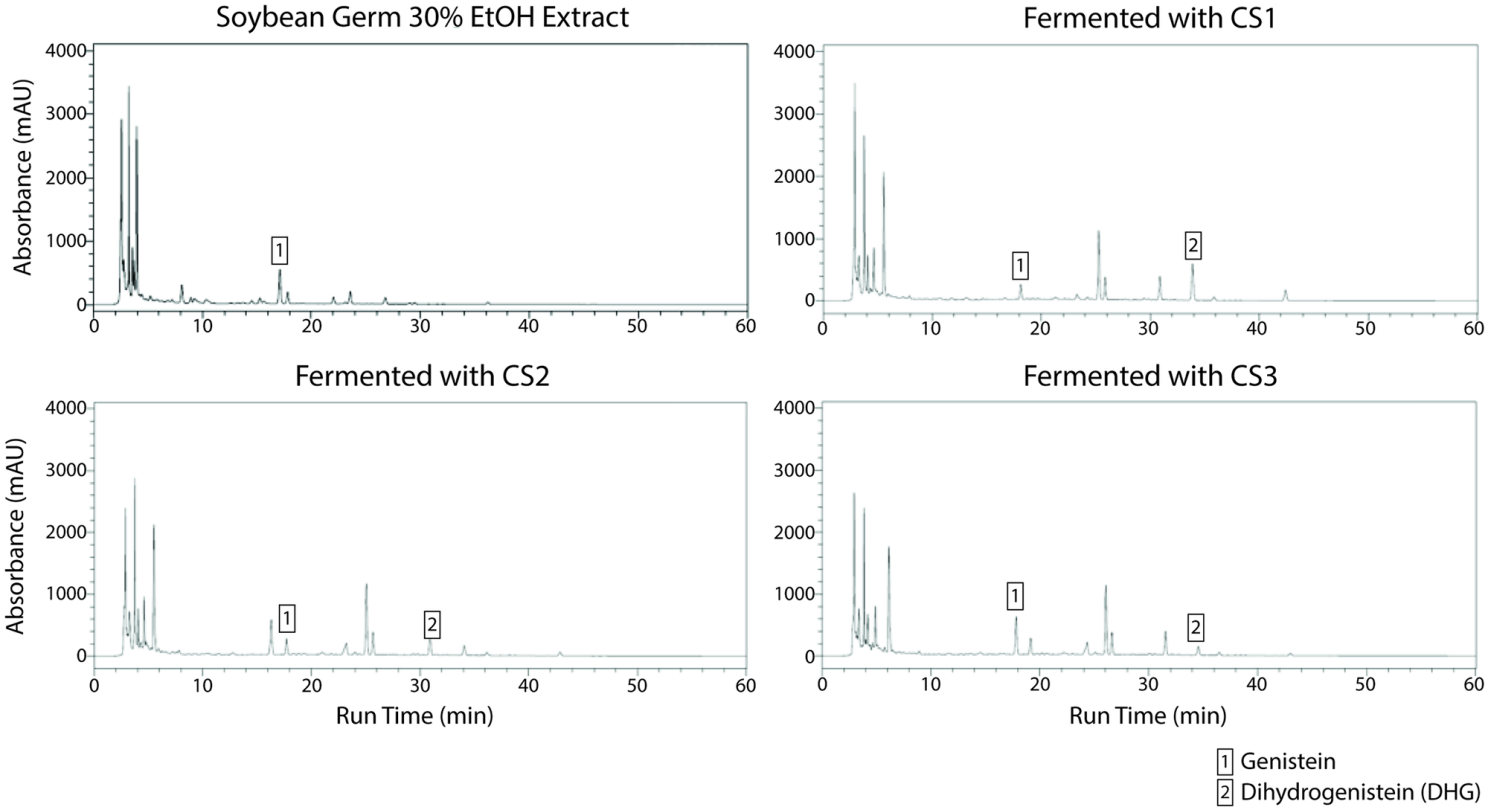
Analysis of equol-related components in soybean germ extract medium before and after fermentation. HPLC analysis of genistein and dihydrogenistein in soybean germ extract medium before and after fermentation.

Soybean germ was chosen for the soy-based extract because soy germ is a significant source of bioactive phytochemicals [23]. HPLC analysis showed that the soybean germ extract had genistein, another equol precursor, but no dihydrogenistein (DHG), which is the next intermediate for equol production. After fermenting the extract with CS1, CS2, and CS3 at 28°C for 24 hr, the amount of genistein decreased, and the amount of DHG increased (Fig 3, Table 3).

**Table 3:** Concentration of genistein and DHG in soybean germ extract medium before and after fermentation.

	Soybean extract (mg/g)	CS1 (mg/g)	CS2 (mg/g)	CS3 (mg/g)
Genistein	135.7	2.1	129.3	131.6
Dihydrogenistein	N/D	122.8	34.3	31.8

However, no equol was found in the fermentation samples from any of the bacterial strains. This result indicates that those strains can produce equol and its related intermediate, DHD, from *P*. *lobata* root extract but not from soybean germ extract. Among the three strains, CS1 had the best ability to produce DHG from genistein.

### Modularity effect of fermentation mixture to estrogen receptor

**Fig 4:**
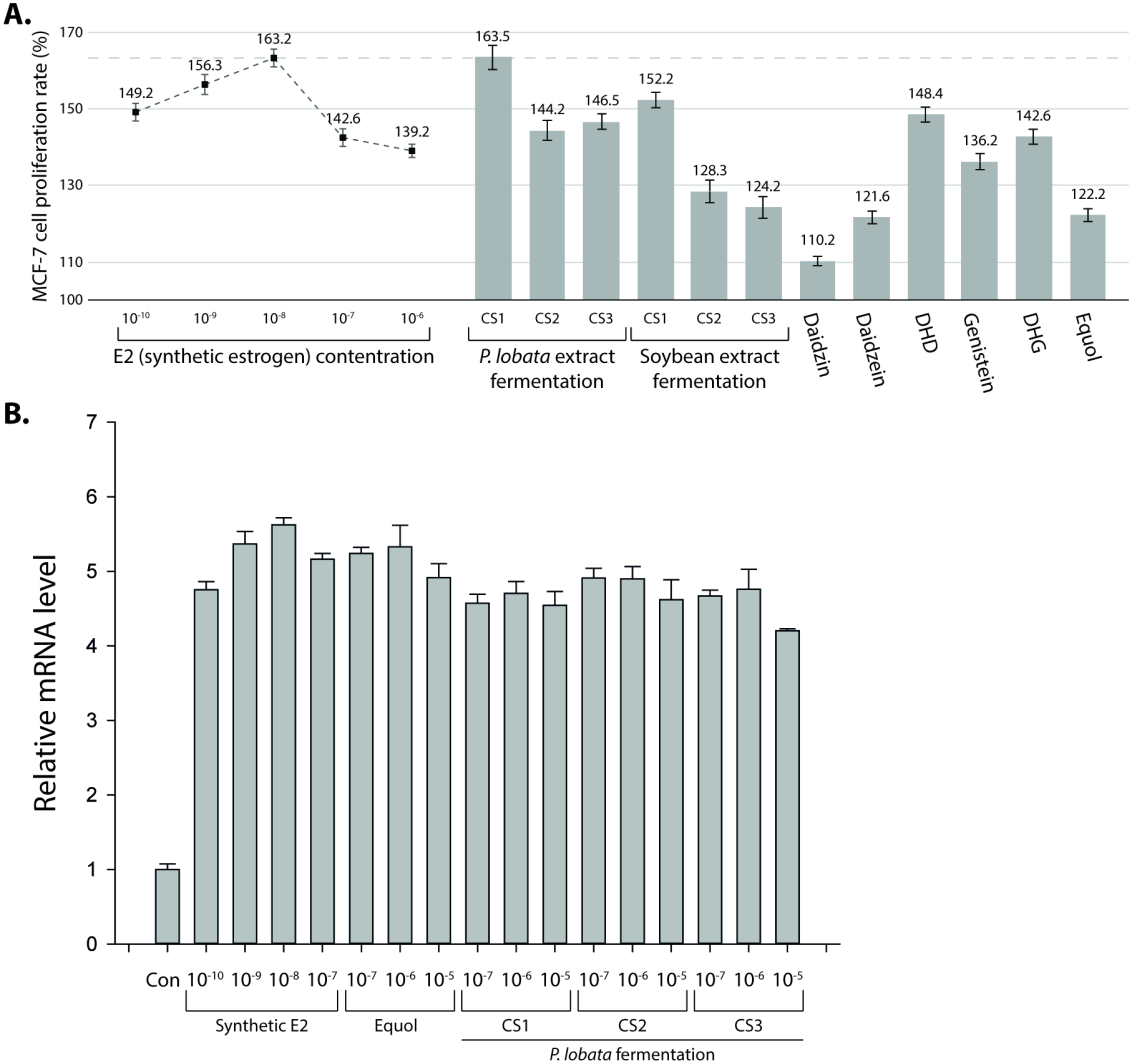
The modularity effect of the fermentation mixture. (A) MCF-7 cell proliferation assay shows the modularity effect of the fermentation mixture. (B) RT-qPCR experiment shows the expression change of the estrogen receptor. The relative mRNA level was normalized to the level of the control.

To measure the modularity effect in the *P*. *lobata* extract fermentation, MCF-7 cell proliferation assay was performed. The *in vitro* MCF-7 cell proliferation assay has been widely used to measure estrogenic activity of molecules [24]. As a control, synthetic estrogen (E_2_, 17 β-estradiol) at different concentrations was used (Fig 4). Synthetic estrogen at 10^−8^ μg/ml induced the most proliferation. Above this concentration, the proliferation rate decreased as the synthetic estrogen concentration increased, indicating that higher E2 concentrations were toxic to the cells. The proliferation rate was also measured with the *P*. *lobata* fermentation mixture, the soybean fermentation mixture, daidzin, daidzein, DHD, genistein, DHG, and equol. Equol-related intermediates, such as DHD, genistein, and DHG, had a better modularity effect than equol itself.

In order to provide an independent line of evidence showing the modularity effect of fermentation mixture with three newly isolated strains, RT-qPCR experiment was performed to measure the transcriptional expression of the estrogen receptor (Fig 4B). The higher level of the estrogen receptor could serve as a proxy of a better modularity effect. As observed from MCF-7 cell proliferation assay, treatment of the synthetic estrogen, and equol increased the expression of the estrogen receptor. Like the synthetic estrogen and equol, the fermentation mixture also increased the expression of the estrogen receptor, suggesting the modularity effect.

Fermentation by CS1, CS2, and CS3 had a modularity effect on the estrogen receptor. Among the three strains, CS1 produced a fermentation mixture with the highest effect, which was higher than that of equol or related intermediates. The *P*. *lobata* fermentation mixture had a higher modularity effect than did the soybean fermentation mixture. These results are particularly interesting because equol and its related intermediates have been more extensively investigated in soy materials. The modularity effect of *P*. *lobata* extract fermented with CS1 was similar to 10^−8^ μg/ml E_2_ in the MCF-7 cell proliferation assay.

## Discussion

In this study, kudzu arrowroot (*Pueraria lobata*) instead of soy-based materials which have been more widely investigated for isoflavones, was used to find bacterial strains that can produce equol or related intermediates from their precursors under aerobic conditions. TLC screening experiments with human feces were used to isolate three new bacterial strains, CS1, CS2, and CS3, that can convert isoflavones in *P*. *lobata* root extract into equol. The amount of equol and DHD produced was measured by HPLC analysis, and CS1 was the most active. In fermentation experiments with soybean germ extract, the strains did not produce equol, but created DHG, which is an equol precursor. Fermenting *P*. *lobata* or soybean germ extract with the three strains had a higher modularity effect than did pure equol in a majority of cases.

The primary goal of this study was to isolate and identify new bacterial strains that could produce equol from *P*. *lobata* extract under aerobic conditions. HPLC analysis showed that these strains produced a reasonable amount of equol or DHD. Previous studies demonstrated that bioconversion to make isoflavones principally depends on the fermentation conditions including the fermentation temperature, time, medium, and starter culture [25]. In this study, fermentation was only performed under one condition, thus the fermentation process could be further optimized by altering oxygen concentration, pH, nitrogen or carbon source supplementation, different base medium, fermentation temperature, or fermentation time. Particularly, strain CS1 produced the most abundant amount of equol and related intermediates; thus, this strain would be the best candidate for process optimization.

During fermentation with soybean germ extract, HPLC analysis revealed that the amount of genistein decreased. This result implies that genistein could be converted into other forms of isoflavones other than DHG. The fermented extract showed a significant peak at around 25 min that could be further be isolated and be analyzed to identify a new isoflavone or their intermediate.

Particularly, using an under-appreciated fermentation material, *P*. *lobata* extract, led to the isolation and identification of new equol-producing bacterial strains. Thus, fermentation of other legumes with the bacterial strains identified in this study could identify new functional molecules.

MCF-7 cell proliferation assay showed that the fermentation mixture, which contains equol and intermediates including DHD, had a much higher modularity effect than did equol itself. Thus, fermentation with new materials and identification of new fermentation products could result in the discovery of functional molecules or mixtures.

## Supporting information

S1 Table ITS sequences for CS1, CS2 and CS3. (DOC)

S2 Table Primers used in this study. (DOC)
