# Supplementary material for: Isolation and identification of new bacterial stains producing equol from Pueraria lobata extract fermentation
Source: PLoS One. 2018 Feb 15;13(2):e0192490. doi: 10.1371/journal.pone.0192490 (PMC5813953; doi:10.1371/journal.pone.0192490)
Supplement: S1 Table — (DOC) [file pone.0192490.s001.doc]

**S1 Table .** ITS sequences for CS1, CS2 and CS3

| Strain | ITS sequences |
| --- | --- |
| CS1 | ACTTCCGTTAATTGATTATGANGTACTTGTACTGATTGAGATTTTAACACGAAGTGAGTGGCGAACGGGTGAGTAACACGATGGGTTAACCTGCCCAGAAGTAGGGGATAACACCTGGAAACAGATGCTAATACCGTATAACAGAGAAAACCGCATGGTTTTCTTTTAAAAGATGGCTCTGCTATCACTTCTGGATGGACCCGCGGCGTATTAGCTAGTTGGTGAGGTAAAGGCTCACCAAGGCAGTGATACGTAGCCGACCTGAGAGGGTAATCGGCCACATTGGGACTGAGACACGGCCCAGACTCCTACGGGAGGCAGCAGTAGGGAATCTTCCACAATGGACGCAAGTCTGATGGAGCAACGCCGCGTGAGTGAAGAAGGGTTTCGGCTCGTAAAGCTCTGTTGTTAAAGAAGAACGTGGGTAAGAGTAACTGTTTACCCcAGTGACGGTATTTAACCAGAAAGCCACGGCTAACTACGTGCCAGCAGCCGCGGTAATACGTAGGTGGCAAGCGTTATCCGGATTTATTGGGCGTAAAGCGAGCGCAGGCGGTCTTTTAAGTCTAATGTGAAAGCCTTCGGCTCAACCGAAGAAGTGCATTGGAAACTGGGAGACTTGAGTGCAGAAGAGGACAGTGGAACTCCATGTGTAGCGGTGAAATGCGTAGATATATGGAAGAACACCAGTGGCGAAGGCGGCTGTCTGGTCTGCAACTGACGCTGAGGCTCGAAAGCATGGGTAGCGAACAGGATTAGATACCCTGGTAGTCCATGCCGTAAACGATGATTACTAAGTGTTGGAGGGTTTCCGCCCTTCAGTGCTGCAGCTAACGCATTAAGTAATCCGCCTGGGGAGTACGACCGCAAGGTTGAAACTCAAAAGAATTGACGGGGGCCCGCACAAGCGGTGGAGCATGTGGTTTAATTCGAAGCTACGCGAAGAACCTTACCAGGTCTTGACATCTTCTGACAGTCTAAGAGATTAGAGGTTCCCTTCGGGGACAGAATGACAGGTGGTGCATGGTTGTCGTCAGCTCGTGTCGTGAGATGTTGGGTTAAGTCCCGCAACGAGCGCAACCCTTATTACTAGTTGCCAGCATTAAGTTGGGCACTCTAGTGAGACTGCCGGTGACAAACCGGAGGAAGGTGGGGACGACGTCAAATCATCATGCCCCTTATGACCTGGGCTACACACGTGTTACAATGGATGGTACAACGAGTCGCGAGACCGCGAGGTTAAGCTAATCTCTTAAAACCATTCTCAGTTCGGACTGTAGGCTGCAACT |
| CS2 | TGGGGGATAACATTTGGAAACAGATGCTAATACCGCATAGATCCAAGAACCGCATGGATTCTTGGCTGAAAGATGGCGTAAGCTATAGCTTTTGGATGGACCCGCGGCGTATTAGCTAGTTGGTGAGGTAATGGCTCACCAAGGCGATGATACGTAGCCGAACTGAGAGGTTGATCGGCCACATTGGGACTGAGACACGGCCCAAACTCCTACGGGAGGCAGCAGTAGGGAATCTTCCACAATGGACGCAAGTCTGATGGAGCAACGCCGCGTGAGTGAAGAAGGCTTTCGGGTCGTAAAACTCTGTTGTTGGAGAAGAATGGTCGGCAGAGTAACTGTTGTCGGCGTGACGGTATCCAACCAGAAAGCCACGGCTAACTACGTGCCAGCAGCCGCGGTAATACGTAGGTGGCAAGCGTTATCCGGATTTATTGGGCGTAAAGCGAGCGCAGGCGGTTTTTTAAGTCTGATGTGAAAGCCCTCGGCTTAACCGAGGAAGCGCATCGGAAACTGGGAAACTTGAGTGCAGAAGAGGACAGTGGAACTCCATGTGTAGCGGTGAAATGCGTAGATATATGGAAGAACACCAGTGGCGAAGGCGGCTGTCTGGTCTGTAACTGACGCTGAGGCTCGAAAGCATGGGTAGCGAACAGGATTAGATACCCTGGTAGTCCATGCCGTAAaCGATGaATGCTAGGTGTTGGAGGGTTTCCGCCCTTCAGTGCCGCAGCTAACGCATTAAGCATTCCGCCTGGGGAGTACGACCGCAAGGTTGAAACTCAAAGGAATTGACGGGGGCCCGCACAAGCGGTGGAGCATGTGGTTTAATTCGAAGCAACGCGAAGAACCTTACCAGGTCTTGACATCTTTTGATCACCTGAGAGATCAGGTTTCCCCTTCGGGGGCAAAATGACAGGTGGTGCATGGTTGTCGTCAGCTCGTGTCGTGAGATGTTGGGTTAAGTCCCGCAACGAGCGCAACCCTTATGACTAGTTGCCAGCATTTAGTTGGGCACTCTAGTAAGACTGCCGGTGATAAACCGGAGGAAGGTGGGGATGACGTCAAATCATCATGCCCCTTATGACTTGGGCTACACACGTGTTACAATGGATGGTAC |
| CS3 | GGGACTGAGACACGGCCCAGACTCCTACGGGAGGCAGCAGTAGGGAATCTTCCACAATGGACGAAAGTCTGATGGAGCAACGCCGCGTGAGTGAAGAAGGTTTTCGGATCGTAAAACTCTGTTGTTGGAGAAGAATGTATCTGATAGTAACTGATCAGGTAGTGACGGTATCCANCCAGAAAGCCACGGCTAACTACGTGCCACCAGCCGCGGTAATACGTAGGTGGCAAGCGTTGTCCGGATTTATTGGGCGTAAAGCGAGCGCAGGCGGTTTCTTNAGTCAGATGTGAAAGCCTTCGGCTCAACCGAAGAAGTGCATCGGAAACTGGAGTTGCAGCCTACAATCCGAACTNAGAATGGTTTTAAGAGATTAGCTAAACCTCGCGGTCTCGCAACTCGTTGTACCATCCATTGTAGCACGTGTGTAGCCCANGTCATANGGGGNATGATGATTTGACGTCGTCCCCACCTTCCTCCGGTTTGTCACCGGCAGTCTCACTANAGTGCCCAMCTTAATGCTGGCAACTAGTAATAAGGGTTGCGCTCGTTGCGGGACTTAACCCAACATCTCACGACACGAGCTGACGACAACCATGCACCACCTGTCACTTTGTCCCCGAAGGGAAAGCTCTATCTCTAGAGTGGTCAAAGGATGTCAAGACCTGGTAAGGTTCTTCGCGTTGCTTCGAATTAAACCACATGCTCCACCGCTTGTGCGGGCCCCCGTCAATTCCTTTGAGTTTCAACCTTGCGGTCGTACTCCCCAGGCGGAGTGCTTAATGCGTTAGCTGCGGCACTGAAGGGCGGAAACCCTCCAACACCTAGCACTCATCGTTTACGGCATGGACTACCAGGGTATCTAATCCTGTTTGCTACCCATGCTTTCGAGCCTCAGCGTCAGTTACASACCAGACAGCCRCCTTCGCCACTGGTGTTCTTCCATATATCTACGCATTTCACCGCTACACATGGAGTTCCACTGTCCACTTCTGCACTCAAGTT |
