# Supplementary material for: Isolation and identification of new bacterial stains producing equol from Pueraria lobata extract fermentation
Source: PLoS One. 2018 Feb 15;13(2):e0192490. doi: 10.1371/journal.pone.0192490 (PMC5813953; doi:10.1371/journal.pone.0192490)
Supplement: S2 Table — (DOC) [file pone.0192490.s002.doc]

**S2 Table .** Primers used in this study

| ITS_amplification | Primers |
| --- | --- |
| Forward | 5’-TCCGTAGGTGAACCTGCGG-3’ |
| Reverse | 5’-TCCTCCGCTTATTGATATGC-3’ |

| RT-PCR primers | Forward | Reverse |
| --- | --- | --- |
| ERα | CGCTACTGTGCAGTGTGCAAT | CCTCACAGGACCAGACTCCATAA |
